# Supplementary material for: Improving the efficiency of scale-up and deployment of community health workers in Mali: A geospatial analysis
Source: PLOS Glob Public Health. 2022 Oct 19;2(10):e0000626. doi: 10.1371/journal.pgph.0000626 (PMC10021816; doi:10.1371/journal.pgph.0000626)
Supplement: Attachment — Submitted filename: Response to Reviewers.docx [file pgph.0000626.s001.docx]

**Response to the editor and reviewers’ comments for the manuscript “Improving the efficiency of scale-up and deployment of community health workers in Mali: a geospatial analysis”**

Dear Editor and reviewers,

Thank you for your feedback and opportunity to resubmit a revised manuscript. Please find below line-by-line responses (in blue font) to your feedback.

Best regards,
Nicholas Oliphant (on behalf of the authors)

Note: Page and line numbers referred to in our responses correspond to the “Revised Manuscript with Track Changes”.

**Editor on journal requirements (responses on blue):**

1. Fig 2: please (a) provide a direct link to the base layer of the map used and ensure this is also included in the figure legend; (b) provide a link to the terms of use / license information for the base layer. We cannot publish proprietary or copyrighted maps (e.g. Google Maps, Mapquest) and the terms of use for your map base layer must be compatible with our CC-BY 4.0 license.

Response: Thank you. Fig 2 does not use a base layer. The layers in the map include: 1) raster of the population distribution 2) administrative boundaries 3) hypothetical (modelled) ASC site location 4) catchment of the hypothetical ASC site (30-minute walk) and 5) CSCom/CSRef location. The data sources for layers 1, 2, and 5 are referenced in the manuscript and are openly available either at the location indicated in the reference or via the link to the publicly available repository in the Data Availability Statement. The references for the data underpinning the hypothetical (modelled) ASC site location and catchment areas (layers 3 and 4 mentioned above) are referenced in the manuscript and accessible via the link to the publicly available repository in the Data Availability Statement.

If your map was obtained from a copyrighted source please amend the figure so that the base map used is from an openly available source. Alternatively, please provide explicit written permission from the copyright holder granting you the right to publish the material under our CC-BY 4.0 license.

Response: Thank you. All layers used are openly available.

Please note that the following CC BY licenses are compatible with PLOS license: CC BY 4.0, CC BY 2.0 and CC BY 3.0, meanwhile such licenses as CC BY-ND 3.0 and others are not compatible due to additional restrictions.

If you are unsure whether you can use a map or not, please do reach out and we will be able to help you. The following websites are good examples of where you can source open access or public domain maps:
* U.S. Geological Survey (USGS) - All maps are in the public domain. (http://www.usgs.gov)
* PlaniGlobe - All maps are published under a Creative Commons license so please cite “PlaniGlobe, http://www.planiglobe.com, CC BY 2.0” in the image credit after the caption. (http://www.planiglobe.com/?lang=enl)
* Natural Earth - All maps are public domain. (http://www.naturalearthdata.com/about/terms-of-use/)

2. Please send a completed 'Competing Interests' statement, including any COIs declared by your co-authors. If you have no competing interests to declare, please state "The authors have declared that no competing interests exist". Otherwise please declare all competing interests beginning with the statement "I have read the journal's policy and the authors of this manuscript have the following competing interests:"

Response: Thank you. We have added a Competing Interests Statement and the accompanying ICMJE COI forms.

Please ensure that you refer to Fig 2 in your text as, if accepted, production will need this reference to link the reader to the figure.
Response: Thank you for noticing this. We have added the reference to Fig 2 in the text on page 14.

We have noticed that you have cited Supporting Information files in your manuscript. However, there are no corresponding files uploaded to the submission. Please upload them as separate files with the item type 'Supporting Information'. Please also ensure that each Supporting Information file has a legend listed in the manuscript after the references list.

Response: Thank you for the comment. We have removed mention of “Supporting Information Files”. The relevant files (references from the MSDS which do not have a DOI or permanent publicly available web address) are included in the Public Data Repository: <https://doi.org/10.5281/zenodo.6551988> so that readers may access them.

Please review your reference list to ensure that it is complete and correct. If you have cited papers that have been retracted, please include the rationale for doing so in the manuscript text, or remove these references and replace them with relevant current references. Any changes to the reference list should be mentioned in the rebuttal letter that accompanies your revised manuscript. If you need to cite a retracted article, indicate the article’s retracted status in the References list and also include a citation and full reference for the retraction notice.

Response: Thank you for the comment. We have reviewed the reference list and made adjustments. We corrected one reference number and added two references (#56 Yang *et al*., and #57 Besada *et al*.) – which required us to update the subsequent reference numbers in the text and reference list. The references are now complete and correct.

Guidelines for resubmitting your figure files are available below the reviewer comments at the end of this letter.

Response: Thank your for the comment. We have used the PACE online tool and resubmitted the Figures after using PACE.

**Reviewer: 1**
**Comments to the Author (responses in blue)**
I hope it to be a fine work of the authors. The study has highlighted the importance of access and coverage of the essential health services. It has attempted to finely present the geospatial analysis on improving the population coverage and distribution of public health services to the unreached communities. It further signifies the importance of Community Health Workers (CHWs) on health service delivery.
Response: Thank you.
Regarding language revisions; the author might need some proof readings. I hope it will sound better to rephrase the first sentence on the introduction as recurrently conjunction "and" has been used.
Response: Thank you for the suggestion. We have proofread the document and revised the first sentence accordingly.

Though the national community health strategy defined the catchment area of a CHW as 3-4 km of the CHW site; what is is the rationale behind considering three populations of interest beyond 5 km of a CSRef or CSCom.

Response: Thank you for the comment and question. CHWs are intended to extend equitable access to community-based primary health care services and reduce morbidity and mortality among mothers and children under-five in communities beyond 5 km of a health facility. Malaria is a main cause of morbidity and mortality among children under-five years of age. The Ministry of Health and Social Development (MSDS is the French acronym) was interested in optimizing scale-up and deployment of CHWs in the context of updates to the national community health strategy and ongoing health sector reform. To this end the MSDS was interested in two policy questions (these are described on page 4). We have revised the text in the Introduction section and moved the policy questions from the Data and Methods section to the Introduction section. We think this helps to frame the analysis and responds to your question.

Please review the citation in the line number 17 of page 15.
Response: Thank you for the comment. We have added an appropriate reference as suggested.

Please include reference for line number 22/23 for any evidence.
Response: Thank you for the comment. We have added appropriate references as suggested.

Besides various limitations of the study; the authors has recommended to be addressed by further researches, i hope the current modeling study could help make better policy decisions regarding the distribution of human resources for health especially at the community levels.

Response: Thank you for the comment. We agree!

**Reviewer: 2**

This is a very resourceful piece for community strategy in primary health care delivery and may be utilized to duly inform decisions around CHW deployment.

Response: Thank you!

It is worth noting however, that mere physical presence of a CHW may not translate to efficiency of care delivery. This is because often these are not people with a background in health training. Their efficacy so much depends on education, day to day training, lived experience and experience working with specific populations. Perhaps, it would have been more useful touching on the level of utilization of the existing CHV network to firm up the rationale for the current study. (How well are we utilizing what we already have before reaching out for what we do not have, which we might not even afford by the way)? Would it benefit the Ministry of health more if it focused on recruiting more CHWs or managing the available CHWs? What has been the opportunity cost (what has the ministry had to forgo in order to cover/take care of the CHW shortage?

In short, if we define shortage in terms of absolute numbers/counts vs. the population then the results for the study are sound. If we look at shortage as a systemic issue that goes beyond just numbers, then we should feel the need to align investments in HRH with the current and future needs of the population and health systems. The scope of the study is well defined, therefore meets its objective. The above recommendations can open room for future research.

Response: Thank you for the thoughtful comment. The Ministry of Health and Social Development (MSDS) has outlined its priorities in the new National Health Sector Development Plan, the health sector reform (Mali Action Plan), and update to the national community health strategy (forthcoming). Developing the priorities entailed various situational analyses, implementation research, and modelling efforts to aid planning with the aim of aligning investments (including for HRH such as CHWs) with current and future needs of the Malian population and the health system, as well as balancing tradeoffs. Previous analyses and implementation research has highlighted the strengths and weaknesesses of CHW performance in Mali and suggested health policy and systems supports to improve performance.^[1]^ Among the MSDS priorities, two are most relevant here. The first is to strengthen CHW performance, efficiency and impact through optimization of health policy and systems support, following WHO normative guidance on the subject and robust evidence from implementation research in Mali on what works to strengthen CHW performance. For example, the Government of Mali recently legally recognized the status of CHWs in Mali as workers within the health system,^[2]^ opening the door for the Government of Mali to progressively take over the costs of payment of CHWs (currently supported by donors) in the context of a long-term sustainable financing pathway. Meanwhile, development partners have committed to accompany the MSDS in its vision by supporting CHWs costs in the interim period while the Government of Mali progressively increases domestic financing for CHWs. Further, on the basis of rigorous implementation research in Mali, the MSDS has prioritized scale-up of a robust CHW supervision model and the use of digital tools to drive CHW performance.^1^ It should be noted that Mali will continue its robust program of implementation research on community health with the aim of fine-tuning future policy and practice and capitalizing on innovation. Thus optimization of CHW performance and analysis of the health policy and planning choices to do so were already covered by other analyses (situational analyses and implementation research) in the context of the strategic planning noted above – and referenced in the paper (e.g., references 54, 55). A second priority of the MSDS is to efficiently expand the CHW network to optimize coverage of the population. This was the subject of our research. We hope this explains the focus of our research. We have adjusted text in the discussion section (page 15) accordingly. We completely agree that both research on CHW performance (and the health policy and systems strengthening to optimize this) AND research on optimizing scale and efficiency of deployment is needed for maximizing impact, efficiency, and sustainability. Our research is intended to serve the needs and vision of the MSDS – which intends to progress on both fronts. And we agree that research is still needed in key areas and will continue to be needed as needs evolve. We hope that our research will be useful, inspire future analyses, and contribute to the culture of continuous improvement and learning that has taken shape in Mali.

1. <https://gh.bmj.com/content/3/2/e000634.abstract>; <https://www.samrc.ac.za/sites/default/files/files/2016-07-11/MaliReport.pdf>; <https://www.ncbi.nlm.nih.gov/pmc/articles/PMC6162089/>; and <https://gh.bmj.com/content/6/11/e007205.abstract>;
2. <https://www.musohealth.org/post/d%C3%A9cret-historique-au-mali-les-asc-au-c%C5%93ur-de-la-r%C3%A9forme-du-syst%C3%A8me-de-sant%C3%A9?lang=fr>
